# The Epidemiological, Clinical, Mycological, and Pathological Features of Rhino-cerebral Mucormycosis: A Systematic Review

**DOI:** 10.30699/IJP.2022.538690.2721

**Published:** 2022-03-08

**Authors:** Mohammadreza Salehi, Shahram Mahmoudi, Omid Rezahosseini, Seyed Jamal Hashemi, Kazem Ahmadikia, Farzad Aala, Nasim Khajavirad, Neda Alijani, Alireza Izadi, Muhammad Ibrahim Getso, Alireza Abdollahi, Arezoo Salami, Seyedeh Rana Khatami, Alireza Adibimehr, Mojtaba Hedayat Yaghoobi, Mohammadmahdi Sabahi, Behshad Pazooki, Farhad Yazdi, Jayran Zebardast, Arash Saifi, Malihe Hasan Nezhad, Masoud Mardani, Sadegh Khodavaisy

**Affiliations:** 1 *Department of Infectious Diseases and Tropical Medicine, Imam Khomeini Hospital Complex, Tehran University of Medical Sciences, Tehran, Iran *; 2 *Department of Parasitology and Mycology, School of Medicine, Iran University of Medical Sciences, Tehran, Iran *; 3 *Viro-immunology Research Unit, Department of Infectious Disease, Rigshospitalet, University of Copenhagen, Copenhagen, Denmark*; 4 *Department of Medical Parasitology and Mycology, School of Medicine, Kerman University of Medical Sciences, Kerman, Iran*; 5 *Department of Parasitology and Mycology, Faculty of Medicine, Kurdistan University of Medical Sciences, Sanandaj, Iran*; 6 *Internal Medicine Department, Imam Khomeini Hospital Complex, Tehran University of Medical Sciences, Tehran, Iran*; 7 *Department of Infectious Diseases, Shariati Hospital, Tehran University of Medical Sciences, Tehran, Iran *; 8 *Department of Medical Microbiology and Parasitology, College of Health Sciences, Bayero University, Kano, Nigeria*; 9 *Department of Pathology, School of Medicine, Imam Khomeini Hospital Complex, Tehran University of Medical Sciences, Tehran, Iran*; 10 *Department of Infectious Diseases, Alborz University of Medical Sciences, Karaj, Iran*; 11 *Student Research Committee, Hamadan University of Medical Sciences, Hamadan, Iran*; 12 *Department of Infectious Diseases, Arak social Security Organization Hospital, Arak, Iran*; 13 *Neurocognitive Science Special Linguistics, Institute for Cognitive Science Studies, Tehran, Iran*; 14 *Infectious Disease and Tropical Medicine Research Center, Shahid Beheshti University of Medical Sciences, Tehran, Iran*

**Keywords:** Cerebral mucormycosis, Diabetes mellitus, Invasive fungal infections

## Abstract

Cerebral mucormycosis (CM) is a life-threatening manifestation of mucormycosis, an angioinvasive fungal infection caused by Mucorales. We sought to systematically review all available case reports to describe epidemiologic features, clinical manifestations, predisposing factors, and diagnostic and treatment strategies of CM. A systematic search was conducted using a combination of the following keywords: "Mucor", "Zygomycetes", "mucormycosis", "cereb*", "brain", "central nervous system", and "intracranial", separately and in combination until December 31^st^ 2018. Data sources included PubMed, Scopus, EMBASE, Web of Science, Science Direct, and Proquest without limiting the time of publication. We included 287 articles corresponding to 345 cases of CM. Out of the 345 cases, 206 (60%) were male with a median age of 44 years; 130 (38%) were reported from North America; 87 (25%) from Asia; and 84 (24%) from Europe. The median time from onset of symptoms to presentation was 3-7 days (65/345, 65%). The highest mortality was observed among patients with diabetes mellitus (*P*=0.003). Debridement of infected brain tissue was associated with improved survival in CM cases (OR 1.5; 95% CI 01.3-1.8; *P*<0.0001). The use of liposomal amphotericin B (L-AMB) was significantly associated with patients' recovery (OR 2.09; 95% CI 1.2-3.4; *P*=0.003). The combination of L-AMB and posaconazole (12.5%) was more effective than the monotherapy treatment of CM cases (*P*=0.009). Clinicians should consider DM as an important risk factor for CM. Moreover, surgical debridement and antifungal combination therapy could be an effective approach in the management of CM patients.

## Introduction

Mucormycosis is a devastatingly angioinvasive fungal infection caused by filamentous fungi belonging to the order Mucorales (1). Mucormycosis is being sporadically reported; however, its aggressive nature, the increasing number of susceptible individuals, the challenge in diagnosis, and associated high mortality would be possible reasons of great concern to the epidemiologists and clinicians (2-5). Some of the classically described predisposing factors for developing mucormycosis are diabetes mellitus (DM), hematological malignancies (HM), hematopoietic stem cell transplantation (HSCT) or solid organ transplant (SOT) recipients, those receiving deferoxamine therapy, malnourished indivi-duals, and injection drug users (IDUs) (4, 6, 7). Mucor-mycosis invades various tissues and can present as rhino-orbito-cerebral, pulmonary, cutaneous, gastrointestinal, and disseminated infection (4). In contrast, rhino-orbito-cerebral mucormycosis (ROCM) is the most common presentation among diabetic patients; the HM patients and HSCT recipients usually present with pulmonary symptoms (4, 8). Cerebral mucormycosis (CM), especially in patients with diabetic ketoacidosis (DKA) or profound neutropenia, can develop both via direct invasion from sino-orbital infection or hematogenously from an infected organ such as lungs (9, 10). Currently, a paucity of information regarding clinical manifestations and predisposing factors affect the diagnosis and treatment of CM (9). We systematically reviewed previously reported cases of mucormycosis to provide a more clear perspective on epidemiologic features, clinical manifestations, radiographic features, predisposing factors, diagnosis, treatment modalities, and outcome of CM.

## Material and Methods

**Fig 1:**
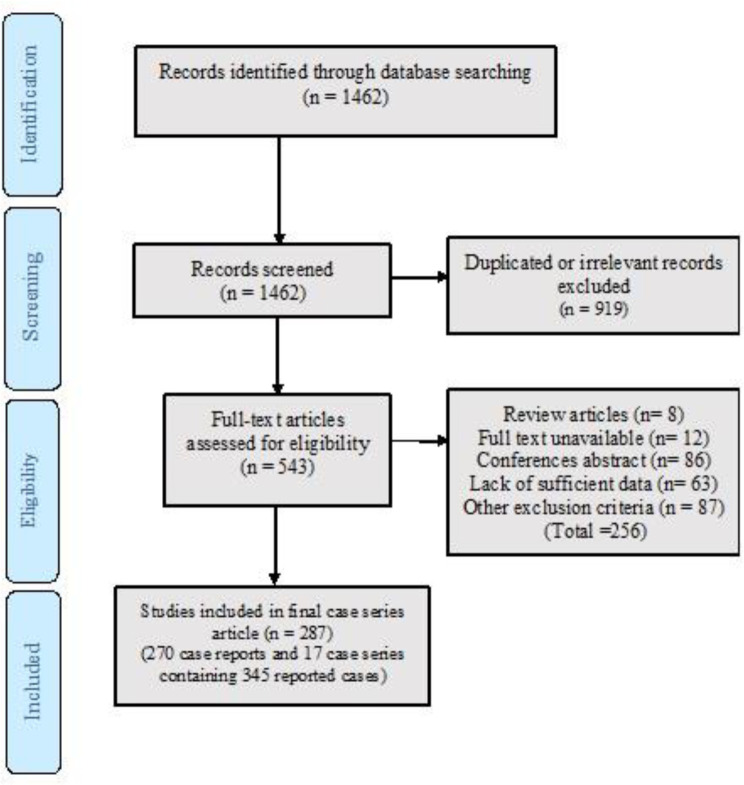
The flow diagram for the included studies

In this study, we followed the preferred reporting items for systematic reviews and meta-analysis (PRISMA) guidelines (10). We designed the clinical question according to the PICO process and selected keywords to cover the question. Figure 1 depicts a flow diagram describing the different phases of this systematic review. To find related articles, a compre-hensive literature review was conducted in global databases such as PubMed, Scopus, EMBASE, Web of Science (WOS), Science Direct, and Proquest without limiting the publication time until 31^st^ December 2018. The search process was accomplished using the following keywords: (((((mucor) OR mucor*) OR zygomycosis) OR zygomyc*)) AND (((((cerebral) OR brain) OR central nervous system) OR intracranial) OR cereb*) both separately and in combination. We also did a manual search in google using the mentioned keywords and selected any additional relevant studies.

Two researchers (M.S and S.K) independently searched the mentioned databases and screened articles. Only case reports or case series in the English literature were included. CM was defined as an invasive mold infection with histopathological or mycological evidence plus the presence of radiological features of brain involvement. Review articles, conference proceedings, and other studies that did not fulfill the inclusion criteria were not included. 

The quality of the included articles was assessed using strengthening the reporting of observational studies in epidemiology (STROBE) checklist; accor-dingly, low quality and irrelevant papers (<10.5) were excluded (11). The quality assessment of the studies was performed independently by two reviewers (S.K and S.M), and disagreements were resolved by the third researcher (M.S).

The rticles by their titles were screened and the full text of relevant articles were reviewed. Afterward, the data containing the name of the first author, year of the publication, country, age, gender, comorbidities, background diseases, clinical symptoms, sources of infection, duration of symptoms, prescribed drugs, surgical procedures, follow up duration, samples types, diagnosis methods, CNS imaging results, antifungal treatment drugs, and outcome were extracted.

A meta-analysis using the STATA software ver. 11 (STATA Corp, College Station, TX, USA) was performed. Proportions were presented as percentages and continuous data as means with standard deviations (SD). A multiple logistic regression was done, and the results were reported as odds ratios (ORs) with a 95% confidence interval (CI). P-value≤0.05 was considered statistically significant**.**

## Results

In the initial search, 1462 articles were retrieved; 1175 articles were excluded according to the exclusion criteria; 287 articles containing 345 cases of CM were included in the meta-review (Figure 1). 

**Epidemiological Features**

**Fig. 2:**
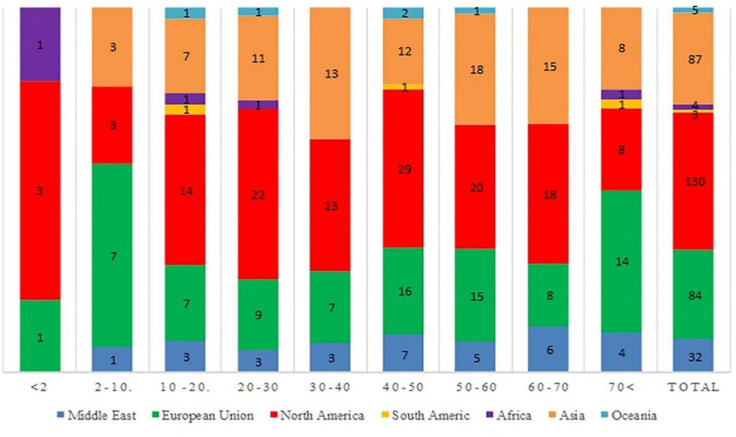
Distribution of 345 reported cases of cerebral mucormycosis according to the age group and geographic region

Two-hundred and six (60%) out of 345 reported CM cases were males with a median age of 44 years (range, 2 months-89 years). Cases were reported from 47 countries, with 130 (38%) from North America, 87 (25%) from Asia, and 84 (24%) from Europe (Figure 2). Epidemiological features and underlying conditions plus results of the multivariable logistic regression in cases who survived or died following CM are shown in Table 1. Seventy-nine (23%) reported cases were immunocompetent and did not receive immunosupp-ressive agents. DM and HMs were reported in 176 (51%) and 93 (26.9%) of the reviewed cases, respectively (Table 1). Among HMs, acute lympho-blastic leukemia (ALL) was reported in 25 (7.2%) cases (*P*<0.001). We divided the published articles according to the publication year (before and after 2000); as such 56% and 47% had DM (*P*=0. 132), while 16% and 30% had HMs (*P*=0.04) before and after the year 2000, respectively. Hypertension, chronic kidney disease, and injection drug use (IDU) were reported in 34 (9.8%), 18 (7.5%), and 18 (5.2%) cases, respectively. 

**Table 1:** Epidemiological features and underlying conditions plus the results of the multivariable logistic regression in cases who survived or died following cerebral mucormycosis

Characteristics	Total n (%) N=345	Survived n (%) N=209	Dead n (%) N=136	OR (95% Cl)	P-value
Underlying conditions					
Alcohol use	10 (2.9)	4 (1.9)	6 (4.4)	1.02 (0.2 -3.7)	**0.970**
Burn	1 (0.3)	0 (0.0)	1 (0.7)	1.65 (1.5-1.8)	**0.419**
Chronic heart disease	11 (3.1)	3 (2.2)	8 (3.8)	0.5 (0.1-2.1)	**0.537**
Chronic kidney disease	32 (9.2)	5 (3.7)	27 (12.9)	0.257 (0.09-.686)	**0.004**
Chronic liver failure	18 (5.2)	3 (2.2)	15 (7.2)	0.2 (0.08-0.1)	**0.04**
Chronic lung disease	15 (4.3)	4 (2.9)	11 (5.3)	0.5 (0.1-1.7)	**0.420**
Concomitant infection	9 (2.6)	2 (1.5)	7 (3.3)	0.43 (0.08-20.1)	**0.492**
Corticosteroid use	37 (10.7)	22 (16.2)	15 (7.2)	2.4 (1.2-5)	**0.008**
Diabetes mellitus with ketoacidosis	14 (23.8)	6 (4.4)	8 (3.8)	1.16 (.393-3.419)	**0.78**
Diabetes mellitus without ketoacidosis	176 (51)	83 (61)	93 (44.5)	1.9 (1.2-3.0)	**0.003**
Hematological malignancy or Hematopoietic stem cell transplantation (HSCT)	80 (23.2)	25 (18.4)	55 (26.3)	0.6 (0.3-1.07)	**0.088**
Solid organ transplantation (SOT)	16 (4.6)	7 (5.1)	9 (4.3)	1.2 (0.4-3.3)	**0.717**
Hypertension	34 (9.8)	13 (9.6)	21 (10.0)	0.9 (0.4-1.9)	**0.88**
Graft versus host disease (GVHD)	11 (3.1)	3 (2.2)	8 (3.8)	0.5 (0.1-2.1)	**0.537**
Injection drug use	18 (5.2)	7 (5.1)	11 (5.3)	0.9 (0.3-2.5)	**0.962**
Other underlying conditions	29 (8.4)	7 (5.1)	22 (10.5)	1.6 (1.5-1.8)	**0.07**
Solid Tumor	6 (1.7)	2 (1.5)	4 (1.9)	0.7 (0.1-4.2)	**0.758**
Trauma	4 (1.2)	3 (2.2)	1 (0.5)	4.6 (0.4-45.5)	**0.304**
Vasculitis	12 (3.4)	2 (1.5)	10 (4.8)	0.297 (0.6-1.37)	**0.135**
Geographical distribution					
Africa	4 (1.2)	1 (0.7)	3 (1.4)	-	**0.694**
Asia	87 (25.2)	30 (22.1)	57 (27.3)		
European Union	84 (24.3)	35 (25.7)	49 (23.4)		
Middle East	32 (9.3)	16 (11.8)	16 (7.7)		
North America	130 (37.7)	50 (36.8)	80 (38.3)		
Oceania	5 (1.4)	2 (1.5)	3 (1.4)		
South America	**3 (0.9)**	**2 (1.5)**	**1 (0.5)**

**Clinical Manifestations**

The time interval between the appearance of clinical signs and symptoms and the first medical visit was reported in 141 cases. The interval was 3-7 days and 8-15 days, respectively, in 65 (46%), and 42 (30%) of the 141 cases. Glasgow coma scale (GCS) ≤10 (coma), 11-14 (lethargy), and 15 (normal) were repor-ted in 13 (3.7%), 40 (11%), and 123 (35%) of the CM cases, respectively. Orbital and neurological signs were reported as the first presentation in 160 (46%) and 142 (41%) of 345 reported cases. Headache, facial signs and symptoms (including swelling, paresthesia, and pain), and fever were reported in 124 (36%), 123 (36%), and 74 (21%) cases, respectively (Table 2). The ethmoid and maxillary sinuses were the most probable primary site of infection reported in 110 (32%) and 107 (31%) of 345 cases. Twenty-four cases (7%) had concurrent lung and brain involvement, among which 5 patients (20.8%) had acute myeloid leukemia (AML), another five (20.8%) had ALL, and 3 patients (12.5%) had DM. Fungemia that was significantly associated with HMs (*P*=0.05) was reported in 5 (1.4%) of the CM cases.

**Radiographic Features**

In brain CT scans or MRIs, a single space-occupying lesion was reported in 98 (28%), brain abscess in 72 (21%), a non-enhancing lesion in 66 (19%), and necrosis in 53 (15%) of reported cases (Table 2). 

**Laboratory Variables**

From blood samples, the white blood cell (WBC) count was reported in 144 (42%) of 345 reported cases. WBC count was <3.5x10^3^ microL in 31 (74%) and >12x10^3^ microL in 72 (20%) of 144 cases. Information about cerebrospinal fluid (CSF) variables was not reported in all reported cases. WBC count in CSF was higher than 3 cells/microL in 37 (95%) out of 39 cases, low glucose (lower than 40 mg/dl) was reported in 5 (13%) out of 39 cases, and high CSF protein (higher than 50 mg/dL) was reported in 25 (57%) out of 44 reported cases.

**Table 2:** Clinical manifestations, site of involvement on imaging, duration of symptoms, and imaging reports plus the results of the multivariable logistic regression in cases who survived or died following cerebral mucormycosis

Characteristics	Total n (%) N= 345	Survived n (%) N=209	Dead n (%) N=136	OR (95% Cl)	P-value
Clinical manifestations					
Auricular symptoms	7 (2)	3 (2.2)	4 (1.9)	1.1 (0.2-5.2)	**0.852**
Facial symptoms (including swelling, paresthesia, and pain)	123 (35.6)	45 (33.1)	78 (37.3)	0.8 (0.5-1.3)	**0.422**
Fever	74 (21.4)	26 (19.1)	48 (23)	0.4 (0.4-1.3)	**0.395**
Headache	124 (35.9)	49 (36.0)	75 (35.9)	1 (0.6-1.57)	**0.978**
Meningismus	2 (0.5)	0 (0)	2 (1)	1.6 (1.5-1.8)	**0.156**
Neurological symptoms	142 (41)	44 (32.4)	98 (46.9)	0.5 (0.3-0.8)	**0.007**
Nasal and oral cavities	60 (17.3)	26 (19.1)	34 (16.3)	1.2 (0.6-2.1)	**0.495**
Orbital symptom	160 (46.3)	69 (50.7)	91 (43.5)	1.3 (0.8-2)	**0.190**
Other clinical manifestations	15 (4.3)	7 (5.1)	8 (3.8)	1.3 (0.4-3.8)	**0.751**
Respiratory symptom	20 (5.7)	6 (4.4)	14 (6.7)	0.634 (0.2-1.7)	**0.366**
Seizure	3 (0.8)	2 (1.5)	1 (0.5)	3.1 (0.2-34.5)	**0.564**
Sites of involvement in radiographic imaging					
Basal gangelia	36 (10.4)	13 (9.6)	23 (11)	0.8 (0.4-1.7)	**0.668**
Bilateral	6 (1.7)	2 (1.5)	4 (1.9)	0.7 (0.81-4.2)	**0.758**
Cavernous Sinus	20 (5.7)	11 (8.1)	9 (4.3)	1.9 (0.7-4.8)	**0.142**
Cranial artery	9 (2.6)	1 (0.7)	8 (3.8)	0.1 (0.02-1.5)	**0.090**
Intra cranial artery	11 (3.1)	6 (4.4)	5 (2.4)	1.8 (0.5-6.2)	**0.297**
Mid brain and pons	18 (5.2)	3 (2.2)	16 (7.7)	0.2 (0.07-0.9)	**0.03**
Multiple cerebral lobes	100 (28.9)	40 (19.1)	59 (13.3)	1.7 (0.7-2.6)	**0.227**
Occipital	17 (4.9)	4 (1.9)	13 (6.2)	0.4 (0.1-1.4)	**0.209**
Parietal	33 (9.5)	11 (8.1)	22 (10.5)	0.7 (0.3-1.5)	**0.452**
Periventricular	11 (3.1)	6 (4.4)	5 (2.4)	1.8 (0.5-6.2)	**0.297**
Temporal	54 (15.6)	21 (15.4)	32 (15.3)	1.0 (0.5-1.8)	**0.974**
Others	5 (1.4)	3 (2.2)	2 (1)	2.3 (0.3-14.1)	**0.38**
Duration of symptoms					
˂3 days	27 (7.8)	9 (6.6)	18 (8.6)	0.7 (0.3-1.7)	**0.545**
3-7 days	65 (18.8)	21 (15.4)	44 (21.1)	0.6 (0.3-1.2)	**0.208**
8-15 days	42 (12.2)	22 (16.2)	20 (9.6)	1.8 (0.9-3.4)	**0.09**
16-30 days	39 (11.3)	17 (12.5)	22 (10.5)	1.2 (0.6-2.3)	**0.572**
˃30	31 (9)	12 (8.8)	19 (9.1)	0.6 (0.4-2)	**0.932**
Imaging report					
Abscess	72 (20.9)	38 (27.9)	34 (16.3)	1.9 (1.2-3.3)	**0.009**
Bleeding	25 (7.2)	0 (0)	25 (12)	1.7 (1.5-1.9)	**0.000**
Cerebritis	3 (0.9)	1 (0.7)	2 (1)	0.7 (0.06-8.5)	**0.827**
Enhancement without abcsess	66 (19.1)	31 (22.8)	35 (16.7)	1.4 (0.8-2.5)	**0.165**
Meningitis	5 (1.4)	2 (1.5)	3 (1.4)	1 (0.1-6.2)	**0.979**
Multiple	53 (15.4)	14 (10.3)	39 (18.7)	0.5 (0.2-0.9)	**0.04**
Necrosis	53 (15.4)	8 (5.9)	45 (21.5)	0.2 (0.1-0.5)	**0.000**
Nodule	8 (2.3)	5 (3.7)	3 (1.4)	2.6 (0.6-11.1)	**0.272**
Single	**98 (28.4)**	**48 (35.1)**	**50 (23.9)**	**1.7 (1.1-2.7)**	**0.028**

**Histopathologic Features and Culture**

Histopathologic examination or culture was performed in 298 (86%) out of 345 reported cases. In comparison, both histopathologic examination and culture were reported in 111 (32%) of 345 cases. Mucorales detection from the histopathological examination was positive in 140 (47%), 87 (29%), 54 (18%), and 17 (5.7%) of 298 cases among the samples stained with hematoxylin and eosin (H&E), Grocott methenamine silver (GMS), periodic acid Schiff (PAS), and other staining methods. The diagnosis was largely reliant on conventional phenotypic techniques after 2000 (*P*=0.04). The culture was performed in 138 (46%) out of 298 cases, with 5 (3.8%) out of 138 cultures being negative. Both histopathology and culture were the diagnostic evidence in 111 (32.2%) of CM cases, while diagnosis using histopathology or culture alone was observed in 298 (86.4%) and 138 (40%), respectively. The use of molecular techniques for CM diagnosis (22/345, 6.3%) was increasingly observed after the year 2000 (*P*<0.001). In all the studied cases, 133 Mucorales fungi were identified. Whereas *Rhizopus* spp., *Lichtemia* spp., *Rhizomucor* spp., and *Mucor* spp. were respectively cultured in 87 (65%), 11 (8.3%), 10 (7.5%), and 8 (6%) of 133 culture-positive cases. Among the 87 cases diagnosed with *Rhizopus *infection, 31 (36%) were *Rhizopus arrhizus,* and 4 (4.6%) were *Rhizopus microspores* being the species identified. 

**Outcome and Treatment**

Death was reported in 209 (60.9%) patients, of which 93 (52.8%, *P*=0.003) was among DM patients and 56 (26.8%) among patients who had HM (*P*=0.1). Surgical debridement was performed in 199 (58%) of 345 cases. The survival OR was 1.5 in CM cases who underwent debridement (95% CI 1.3-1.8; *P*<0.0001). The antifungal therapy with amphotericin B-deoxy-cholate (AmB-d), liposomal amphotericin B (L-AMB), or posaconazole was documented in 186 (54%), 87 (25%), 37 (11%) patients, respectively. The survival OR was 2.09 in patients who received L-AMB (95% CI 1.2-3.4; *P*=0.003). Combination therapy with L-AMB and posaconazole was reported in 45 (13%) out of the 348 cases. There was a significant relation (*P*=0.009) between survival and L-AMB-posaconazole combination therapy (12.5% survivors vs. 5.3% dead among cases who had received the combined antifungal regimen; this relationship was not significant for other combined antifungal regimens).

**Table 3:** Prescribed antifungals in cases of cerebral mucormycosis plus the results of the multivariable logistic regression in cases who survived or died following cerebral mucormycosis

P-value	OR (95% Cl)	Dead n (%) N=136	Survived n (%) N=209	Total n (%) N=345	Antifungals
**0.554**	1.4 (0.7-1.7)	76 (55.9)	110 (52.6)	186 (53.9)	Amphotericin B-Deoxycholate
**0. 900**	1.6 (1.5-1.8)	-	1 (0.4)	1 (0.3)	Anidulafungin
**0. 005**	2.5 (2.2-2.9)	5 (3.6)	-	5 (1.4)	Caspofungin
**0. 553**	0.6 (0.1-3.1)	2 (1.4)	5 (2.3)	7 (2)	Fluconazole
**0.942**	1.0 (0.3-2.9)	5(3.7%)	8(3.8%)	13 (3.8)	Flucytosine
**0.900**	1.5 (2.1-11.9)	2 (1.4)	2 (0.9)	4 (1.2)	Isavuconazole
**0. 037**	2.4 (1.2-5)	6 (4.4)	2 (0.9)	8 (2.3)	Itraconazole
**0.900**	1.5 (0.2-11.09)	2 (1.4)	2 (0.9)	4 (1.3)	Ketoconazole
**0. 003**	2.09 (1.2-3.4)	46 (33.8)	41(19.6)	87 (25.2)	Liposomal Amphotericin B
**0.758**	0.7 (0.1-4.2)	2 (1.4)	4 (1.9)	6 (1.7)	Micafungin
**0. 9**	1.6 (1.5-1.8)	-	1 (0.4)	1 (0.3)	Nystatin
**0. 008**	2.49 (1.2-5.00)	22 (16.1)	15 (7.1)	37 (10.7)	Posaconazole
**0. 758**	**0.7 (0.1-4.2)**	**2 (1.4)**	**4 (1.9)**	**6 (1.7)**	Voriconazole

## Discussion

Despite the clinical significance of cerebral mucormycosis (12), comprehensive data regarding its epidemiology and clinical manifestations are lacking. Accordingly, the present systematic review was conducted to fill this gap of knowledge. CM is globally distributed, and reports of this infection have been published from 47 countries, with the majority being from North America (130/345, 37.68%). This finding is similar to that of Kerezoudis *et al.* who reported a meta-analysis on isolated cerebral mucormycosis (13) but different from the results of a recent systematic review of mucormycosis case reports that found the majority of reports from Europe (290/851, 34%) followed by Asia (267/851, 31%) (14). This difference might have emanated from different inclusion criteria used in the studies or due to a higher incidence of CM in North America in comparison to other forms of mucormycosis. Furthermore, because the definitive diagnosis of mucormycosis is challenging and more than 90% of cases might have been confirmed by post-mortem examinations (15, 16), in resource-limited and developing countries, where access to diagnostic methods and devices is limited, diagnosis and reporting of CM cases might be difficult. These limitations, in turn, result in underreporting of CM in some geographical regions. 

CM can affect humans in all age groups. In the present study, patients' age spanned a wide range: from 2 months to 89 years with a median of 44.2 years. In another systematic review of isolated CM cases, the mean age of 28 years was reported (13). Moreover, similar to the results of other systematic reviews that reported 57% (13), 58% (17), and 63% (14) of patients being males, in the present study, males (57.9%) constituted a larger proportion of CM cases. Future studies need to be done to determine the exact role of the male gender as a potential risk factor for mucormycosis. Mucormycosis is generally known as an infection affecting patients with underlying conditions. Diabetes has been and is still the most common risk factor for mucormycosis in resource-limited countries (16). 

In the present study, 176 (51%) cases of CM were diabetic and 14 (23.8%) had diabetic ketoacidosis. In recent years, we have witnessed the rise in the number of patients with immunosuppressed conditions such as solid organ/hematopoietic stem cell transplant recipients and those under chemotherapy; these patients are known to be highly vulnerable to mucormycosis (16). In agreement with this statement, a large proportion (n=96, 27.8%) of the reported CM cases belong to these groups. 

In addition to the above-mentioned underlying conditions, a divergent set of other diseases have also been reported among CM patients (Table 1). Surprisingly, in the current study, 22.9% of CM patients were immunocompetent and not receiving any immunosuppressant drugs. A similar finding was reported in another systematic review of mucormycosis cases (14). Accordingly, CM should be taken into account in all patient groups, even among healthy individuals with suspicious signs and symptoms. Orbital (46.4%) and neurological (41.2%) symptoms were the most common presentations in CM patients followed by headache (35.9%). In a review of isolated CM cases (13), altered mental status (54%) and headache (51%) were reported as the common signs and symptoms, which differ from our findings. However, the unspecific signs and symptoms similarly add to the complexity of the diagnosis of CM. Orbital involvement was the most common and interesting symptom reported previously (18), indicating the need to consider cerebral mucormycosis in patients with orbital symptoms. 

As mentioned previously, neurological symptoms are often associated with other extra-cerebral sym-ptoms (19). Although headache was a common clinical sign, fever was reported in just over one-fifth of cases. However, in some studies, up to 50% of patients had a fever (19). Overall, fever does not appear to be a reliable clinical sign to diagnose or rule out this invasive infection. In Kursun *et al.* study*,* cavernous sinus involvement was the most common sign of cerebral imaging in 27 patients followed by brain infarction, while in our study, abscess-like lesions and contrast-enhancing were the most common (18). 

*Rhizopus arrhizus* was dominant *Rhizopus *spp*.* identified to cause CM in this study. Similarly, other reviews on different patient groups have reported *Rhizopus* spp. to be the leading etiologic agents of mucormycosis (13, 14, 17, 20). Despite this agreement between various studies, results regarding the second and third-ranked causes of the disease are contro-versial. Although in the present study, *Lichtemia* spp. and *Rhizomucor* spp. were respectively the second and third common causes of CM, studies focused on all forms of mucormycosis (14, 17, 20) and even another review of isolated CM (13), have reported *Mucor* spp. to be the second common cause of the disease. Based on our results, obtained from a large number of cases, it could be hypothesized that *Lichtemia *spp. have a higher tendency for cerebral invasion in comparison to *Mucor* spp. These findings need to be confirmed by further investigations. 

## Conclusion

In conclusion, based on our findings, CM would be associated with global distribution and might present with a variety of orbital and neurological symptoms. Mucormycosis can invade any part of the brain but shows more predilection to the temporal and frontal lobes. DM and HMs are considered as common risk factors. Moreover, surgical debridement and antifungal combination treatment (L-AMB plus Posaconazole) could be an effective approach in management of the CM patients.

## Authors' Contributions

MS, SK, and OR designed the study. NK, NA, SRK, AA, MHY, MS, FY, SJH, JZ, MIG, and OR performed the initial search and wrote the first draft of the manuscript, KA, SM, SK, FA, and MS cross-checked the referenced articles. SK, SM, and MS summarized the articles. All authors revised and commented on the manuscript. All authors read and approved the final version of the manuscript.

## Conflict of Interest

OR received a grant from the Research Foundation of Rigshospitalet related, and a grant from A.P. MØLLER FONDEN not related to this work. The authors declare that they have no competing interests.
